# Variably Protease-Sensitive Prionopathy, a Unique Prion Variant with Inefficient Transmission Properties

**DOI:** 10.3201/eid2012.140214

**Published:** 2014-12

**Authors:** Abigail B. Diack, Diane L. Ritchie, Alexander H. Peden, Deborah Brown, Aileen Boyle, Laura Morabito, David Maclennan, Paul Burgoyne, Casper Jansen, Richard S. Knight, Pedro Piccardo, James W. Ironside, Jean C. Manson

**Affiliations:** The Roslin Institute, University of Edinburgh, Easter Bush, Scotland, UK (A.B. Diack, D. Brown, A, Boyle, L. Morabito, D. Maclennan, P. Burgoyne, J.C. Manson);; School of Clinical Sciences, University of Edinburgh, Edinburgh, Scotland, UK (D.L. Ritchie, A.H. Peden, R.S. Knight, J.W. Ironside);; Food and Drug Administration, Rockville, Maryland, USA (P. Piccardo);; University Medical Centre Utrecht, Utrecht, the Netherlands (C. Jansen)

**Keywords:** prion, variably protease-sensitive prionopathy, VPSPr, sporadic Creutzfeldt-Jakob disease, sCJD, transmissible spongiform encephalopathy, TSE, prion protein, human prion disease, human-to-human transmission, prions and related diseases

## Abstract

Variably protease-sensitive prionopathy (VPSPr) can occur in persons of all codon 129 genotypes in the human prion protein gene (*PRNP*) and is characterized by a unique biochemical profile when compared with other human prion diseases. We investigated transmission properties of VPSPr by inoculating transgenic mice expressing human *PRNP* with brain tissue from 2 persons with the valine-homozygous (VV) and 1 with the heterozygous methionine/valine codon 129 genotype. No clinical signs or vacuolar pathology were observed in any inoculated mice. Small deposits of prion protein accumulated in the brains of inoculated mice after challenge with brain material from VV VPSPr patients. Some of these deposits resembled microplaques that occur in the brains of VPSPr patients. Comparison of these transmission properties with those of sporadic Creutzfeldt-Jakob disease in the same lines of mice indicated that VPSPr has distinct biological properties. Moreover, we established that VPSPr has limited potential for human-to-human transmission.

## Abstract 2

Transmission properties of this prion disease are biologically distinct, and the disease has limited potential for human-to-human transmission.

Human prion diseases, also called transmissible spongiform encephalopathies, are a group of rare and inevitably fatal neurodegenerative diseases. Prion diseases are unique in that they occur as idiopathic (sporadic), familial, and acquired disorders. The sporadic form of Creutzfeldt-Jakob disease (sCJD) accounts for >80% of all human prion diseases. Six subtypes of sCJD have been classified according to the prion protein (PrP) genotype at codon 129 (methionine [M]/M, M/valine [V], VV) and the biochemical profile of the protease-resistant core of the abnormal disease-specific PrP (PrP^res^) (PrP^res^ type 1 or PrP^res^ type 2A or 2B) (*1*,*2*). Experimental transmission of brain tissue from patients of these 6 different sCJD subtypes into 3 transgenic mouse lines expressing different human prion protein gene *PRNP* sequences (coding for 129MM, MV, and VV) has identified 4 distinct strains of the CJD agent (*3*).

In 2008, a novel prion disease, initially referred to as protease-sensitive prionopathy, was reported in 11 patients who had been referred to the National Prion Disease Pathology Surveillance Center (Cleveland, OH, USA) during May 2002–January 2006. All 11 patients were of the *PRNP* codon 129VV genotype, and postmortem examination of brain tissues showed that the patients had a spongiform encephalopathy (*4*). As with patients with sCJD, these patients had no mutations in the *PRNP* coding region, and no risk factors for the development of iatrogenic CJD were identified among the patients. The defining feature of this group of patients was the unusual biochemical properties of the abnormal PrP in the brain. Compared with the biochemical properties of PrP^res^ in sCJD, the PrP^res^ in VPSPr was found to be much less resistant to protease digestion. VPSPr PrP^res^ shows a faint ladder-like appearance of protease-resistant fragments on Western blot and a prominent low–molecular weight fragment of ≈8 kDa. The neuropathologic features in this group were also unusual, in particular, the accumulation of microplaques within the cerebellum and thalamus, which stained intensely for PrP.

Since the original description of those 11 cases, 19 additional cases have been reported, including some in patients of the *PRNP* codon 129MM and 129MV genotypes (*5*–*7*). Although case numbers remain low, the prevalence of this novel prion disease appears to vary according to the codon 129 genotype of affected persons: 62% of reported cases have been detected in persons of the *PRNP* codon 129VV genotype. In comparison, 17% of sCJD cases and only 12% of the general white population are of the *PRNP* codon 129VV genotype (*8*,*9*). Subsequent studies showed differences between the 3 codon 129 genotypes in protease digestion sensitivity of the abnormal PrP in the brain. This difference in protease sensitivity has resulted in the condition being renamed variably protease-sensitive prionopathy (VPSPr) (*10*).

The presence of PrP^res^ in VPSPr suggests that PrP plays a central role in the disease process. However, the relationship between different forms of PrP and prion disease has not been established. It is possible that the protease-sensitive and the truncated forms of the abnormal PrP may contribute to the unique neuropathology of VPSPr and may also influence the potential for transmission of disease to other persons.

Human-to-human transmission of prion diseases is of great concern for public health reasons (*11*–*13*). The use of gene-targeted transgenic mice expressing human PrP enables the direct comparison of transmission properties by using well-defined strains of sCJD and variant CJD (vCJD). Moreover, this system enables the prediction of disease transmission between persons and has been used extensively to predict the potential for human-to-human spread of sCJD and vCJD (*3*,*14*–*16*). In this study, we challenged human PrP–expressing transgenic mice with brain tissue from 3 persons with VPSPr and directly compared the data with those from previous sCJD transmission experiments in these mouse lines. Thus, we determined whether any biological similarities exist between these apparently different prion diseases. Furthermore, these transmissions enabled an assessment of the potential for human-to-human transmission of VPSPr.

## Methods

### Human Tissues

Frozen brain tissues from 3 patients with VPSPr were investigated in this transmission series. Tissues analyzed were obtained from 2 patients who originated from the United Kingdom: 1 patient had the *PRNP* codon 129VV genotype (patient UK-VV), and the other had the 129MV genotype (patient UK-MV). The third patient originated from the Netherlands and had the *PRNP* codon 129VV genotype (patient NL-VV). The clinical, neuropathologic and PrP biochemical features of these cases have been described in detail elsewhere (*5*,*6*,*17*). Patient details for the 3 cases, including age, sex, neuropathologic features, and PrP^res^ type, are summarized in Table 1. As reference standards in Western blot experiments, we used frozen brain tissues in which the 8-kDa and the 2A PrP^res^ types were readily detectable; the tissues were from patients with typical UK cases of sCJD (subtypes MM1, MM2, VV1, and VV2) and from another UK patient (codon 129VV) with VPSPr.

**Table 1:** Characteristics of patients with variably protease-sensitive prionopathy whose brain samples were selected for transmission studies*

Patient, sex	Age at death, y	Disease duration, mo	Clinical signs and symptoms	Neuropathologic features	PrP^res^ type
NL-VV, M†	57	20	Progressive dementia, spastic paraplegia, sensorimotor polyneuropathy	Mild to moderate spongiform change in basal ganglia, and cerebral and cerebellar cortices. Coarse granular deposits of PrP in cerebral cortex, basal ganglia, and thalamus. PrP microplaques present within molecular layer of cerebellar cortex	Faint ladder-like appearance of protease-resistant fragments with a prominent low–molecular weight fragment
UK-VV, F‡	59	42	Progressive dementia, emotional and obsessive behavior (early), very occasional myoclonus (late)	Mild to moderate spongiform change in basal ganglia, thalamus, and cerebral and cerebellar cortices. Widespread granular accumulations of PrP in all brain regions. PrP microplaques present within molecular layer of cerebellar cortex	Faint, ladder-like appearance of protease-resistant fragments with a prominent low–molecular weight fragment
UK-MV, M§	76	12	Forgetfulness, visuospatial perceptual problems, difficulties walking, action tremor, akinetic mutism	Spongiform change most prominent in the frontal cortex. PrP microplaques, synaptic and granular accumulations of PrP restricted to cerebral cortex, basal ganglia, and thalamus. Diffuse Lewy body and tau pathology observed, with amyloid-β plaques and a widespread amyloid angiopathy	Faint ladder-like appearance of protease-resistant fragments including a low–molecular weight fragment

*PrP, prion protein; PrP^res^, protease-resistant isoform of the disease-specific PrP. †NL-VV, patient from the Netherlands who was homozygous for valine at codon 129 of the PrP gene (*PRNP*). Case report, Jansen et al. (*6*). ‡UK-VV, patient from the United Kingdom who was homozygous for valine at *PRNP* codon 129. Case report, Head et al. (*5*). §UK-MV, patient from the United Kingdom who was heterozygous for methionine/valine at *PRNP* codon 129. Case report, Head et al. (*17*).

Consent and ethical approval for the retention and use of these materials for research was obtained by the Lothian NHS Board Research Ethics Committee (Reference: LREC/2000/4/157). Material was sourced through The Edinburgh Brain Bank (Scotland, UK).

Gray matter–enriched frontal cortex tissue samples (≈250 mg) had been obtained at autopsy from each of the 3 persons in our study. The samples were homogenized at a 10% (wt/vol) concentration in sterile physiologic saline and stored at −20°C until use. Before being inoculated into mice, the homogenates were further diluted to a 1% (wt/vol) concentration in sterile physiologic saline.

### Experimental Animals

Mice from 3 lines of transgenic mice expressing human PrP (designated HuMM, HuMV, and HuVV, according to the *PRNP* codon 129 genotype) were challenged in this transmission series (*15*). Mice were anesthetized and inoculated intracerebrally with 20 μL of a 1% brain homogenate. Beginning on postinoculation day 100, the mice were scored on a weekly basis for clinical signs of neurologic disease, as described by Fraser and Dickinson (*18*). Mice were humanely killed at the clinical endpoint for prion disease or at the end of the animal’s full life span. Incubation periods were calculated as the number of days between brain-tissue inoculation and the clinical endpoint, when mice showed unequivocal neurologic disease. In the absence of an incubation period, the survival time (in days) was calculated. Brains were removed from the mice postmortem and sagittally sectioned; half of the brain was snap-frozen for biochemical analysis, and the other half was fixed in 10% formal saline for histologic analysis. These animal experiments were approved by The Roslin Institute's (University of Edinburgh) Animal Welfare and Ethical Review Committee and conducted according to the regulations of the UK Home Office Animals (Scientific Procedures) Act 1986.

### Scoring of Vacuolation

Mouse brains for histologic analysis were fixed in formal saline for a minimum of 48 h before being immersed in 96% formic acid for 1.5 h to reduce the titer of the infectious agent. Brains were trimmed coronally into 5 standard rostrocaudal levels, resulting in 5 brain slices. Tissues were then embedded in paraffin wax and cut into serial 5-μm sections. A single section from all inoculated mice was stained with hematoxylin and eosin to determine the presence and severity of disease-specific vacuolation in 9 standard gray matter regions and 3 white matter regions, a protocol referred to as lesion profiling (*18*).

### Immunohistochemistry

Immunohistochemical analysis for PrP was performed by using 4 PrP monoclonal antibodies that recognize different residues of the PrP: 1) 3F4/epitope: aa 109–112 (Cambridge Bioscience, Cambridge, UK); 2) 12F10/epitope: aa 142–160 (Bioquote Ltd, York, UK); 3) 6H4/epitope: aa 144–152 (Prionics AG, Schlieren, Switzerland); and 4) monoclonal antibody KG9/aa140–180 (TSE Resource Centre, The Roslin Institute). In brief, 5-μm paraffin-embedded tissue sections were autoclaved at 121°C in distilled water for 10 min and then immersed in 96% formic acid for 10 min. Sections were immersed in proteinase K solution (5 μg/mL) for 10 min and then blocked for 20 min in normal rabbit serum, after which they were incubated overnight at room temperature with the primary antibodies (3F4, 5 μg/mL; 6H4, 500 ng/mL; 12F10, 30 ng/mL; and KG9, 40 ng/mL). After overnight incubation, sections were incubated for 1 h in an anti-mouse biotinylated antibody (Jackson ImmunoResearch Laboratories, Inc., West Grove, PA, USA), and then immunolabeling was completed by using a VECTASTAIN Elite ABC Kit (Vector Laboratories, Burlingame, CA, USA). Staining was then visualized by using 3,3′-diaminobenzidine chromogen.

The presence of gliosis was assessed by incubating tissue sections with Polyclonal Rabbit Anti-Cow Glial Fibrillary Acidic Protein (Dako, Ely, UK) for 1 h at room temperature. Sections were then incubated for 1 h at room temperature with an anti-rabbit biotinylated antibody (Jackson ImmunoResearch Laboratories, Inc.) before the immunolabeling was completed by using a VECTASTAIN Elite ABC Kit. Staining was then visualized by using 3,3′-diaminobenzidine chromogen.

### Thioflavin-S Visualization

Paraffin-embedded tissue sections were immersed in hematoxylin solution for 1 min and rinsed in running water before being immersed in Scott’s tap water for 30 s. Sections were then immersed in 1% Thioflavin-S (Sigma, Gillingham, UK) for 5 min, followed by 3 dips in 70% alcohol. Tissue sections were then rinsed well in water and mounted.

### Biochemical Studies of Brain Samples

The method we used for studying brain tissues was based on our previous Western blotting technique (*19*). For analysis, 10% (wt/vol) brain tissue homogenates were prepared by homogenization of brain material in 9 volumes (wt/vol) of Tris-buffered saline, pH 7.6, containing 0.5% Nonidet P40 and 0.5% sodium deoxycholate. Aliquots of the cleared 10% brain homogenates were subjected to limited proteolysis by digestion with proteinase K (50 μg/mL) for 1 h at 37°C. The reaction was terminated by the addition of Pefabloc SC (Roche, Burgess Hill, UK) to a final concentration of 1 mM/L. Proteinase K–treated and non–proteinase K–treated samples (5 μL) were analyzed by Western blot. Polyacrylamide gel electrophoresis and Western blotting were performed by using NuPAGE Novex 10% Bis-Tris Protein Gels, 1.0 mm, (Life Technologies, Paisley, UK) as previously described (*19*). The gel electrophoresis time was abbreviated to retain low–molecular mass proteins (*5*,*6*). The proteins were transferred onto Hybond-P PVDF membrane (GE Healthcare Life Sciences, Amersham, UK). Immunodetection of PrP was carried out by using monoclonal antibody 3F4 (Millipore, Watford, UK) at a final concentration of 75 ng/mL for 1 h. For comparison, immunodetection of PrP on proteinase K–treated extracts was carried out by using monoclonal antibody 1E4 (provided by J. Langeveld) at a final concentration of 1 μg/mL (*6*). The secondary antibody was ECL Anti-mouse IgG, peroxidase-linked species-specific F(ab′)2 fragment (from sheep) (GE Healthcare Life Sciences), used at a concentration of 1/25,000 for 1 h. Amersham ECL Prime Western Blotting Detection Reagent (GE Healthcare Life Sciences) was used for detection of proteins. The blots were exposed to ECL Hyperfilm (GE Healthcare Life Sciences) for various amounts of time or were analyzed by using the ChemiDoc XRS+ System with Image Lab Software (Bio-Rad, Hemel Hempstead, UK).

## Results

### Biochemical Analysis of Brain Tissue from VPSPr Patients

**Figure 1:**
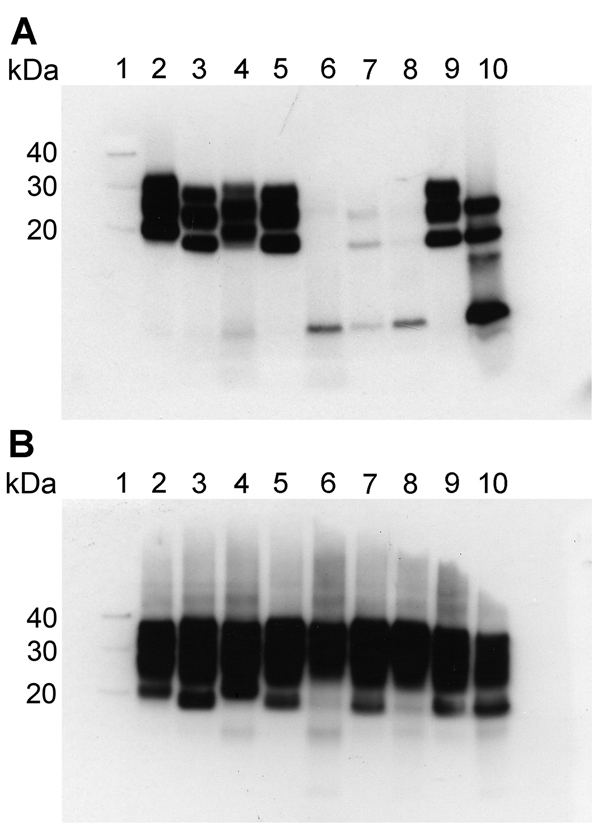
Western blot analysis of protease-resistant isoforms of PrP (PrP^res^) in extracts of frontal cortex tissue prepared from postmortem samples from 4 persons with sCJD and from 3 persons with VPSPr whose brain samples were used for experimental transmission studies in transgenic mice. Results are shown for extracts treated (A) and not treated (B) with proteinase K. All lanes were loaded with 5.0 μL of 10% (wt/vol) brain homogenate, except lanes 9 and 10 in (A), which were loaded with 1.5 μL and 20.0 μL, respectively. Blots were probed with Anti-Prion Protein Antibody monoclonal antibody 3F4 (Millipore, Watford, UK). Lane 1, molecular weight marker (sizes indicated at left in kDa); lane 2, sCJD subtype MM1; lane 3, sCJD subtype MM2; lane 4, sCJD subtype VV1; lane 5, sCJD subtype VV2; lane 6, VPSPr UK-VV; lane 7, VPSPr UK-MV; lane 8, VPSPr NL-VV; lane 9, diagnostic reference sample (sCJD subtype VV2); lane 10, diagnostic reference sample (VPSPr VV). MM, homozygous for methionine; MV, heterozygous for methionine/valine; NL-VV, patient from the Netherlands who had VPSPr and the codon 129VV genotype; sCJD, sporadic Creutzfeldt-Jakob disease; UK-VV and UK-MV, patients from the United Kingdom who had VPSPr and the codon 129VV and 129MV genotypes, respectively; VPSPr, variably protease-sensitive prionopathy; VV, homozygous for valine.

We performed Western blot analysis on extracts of homogenates prepared from brain samples from all 3 VPSPr patients. For comparison, we ran these extracts alongside extracts of homogenates prepared from brain samples from 4 sCJD patients (sCJD subtypes MM1, MM2, VV1, and VV2) representing each of the 4 distinct strains of sCJD agent, as identified by transmission to transgenic mice (*3*). Western blotting was performed with and without proteinase K digestion by using the PrP antibody 3F4 (Figure 1). In the absence of proteinase K digestion, extracts from the 3 VPSPr patients showed a similar relative load of PrP when compared with extracts from sCJD patients (Figure 1, panel B). After the sCJD and VPSPr extracts were digested with proteinase K, their biochemical profiles and PrP^res^ loads differed (Figure 1, panel A). The biochemical profile of the extracts prepared from brain tissue from patients UK-VV and NL-VV VPSPr showed a single low–molecular weight fragment (<10 kDa), characteristic of VPSPr. In the extract from the UK-MV case, this low–molecular weight fragment was detected in addition to a faint ladder-like pattern of PrP^res^ fragment.

**Figure 2:**
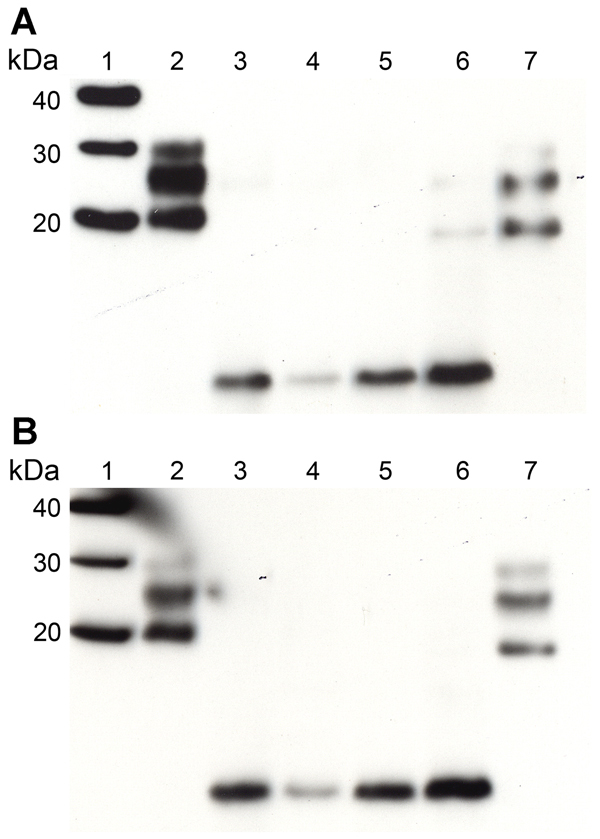
Western blot analysis of PrP^res^ in extracts of frontal cortex tissue prepared from postmortem samples from 2 persons with sCJD (subtypes MM1 and VV2) and the 3 persons with VPSPr whose brain samples were used for experimental transmission studies in transgenic mice (patients NL-VV, UK-VV, and UK-MV). Extracts from another patient who had VPSPr of UK origin (codon 129VV genotype) was also included on the blot (lane 6). Duplicate blots were probed with the following monoclonal antibodies: Anti-Prion Protein Antibody monoclonal antibody 3F4 (Millipore, Watford, UK) (A) and 1E4 (provided by J. Langeveld) (B). The 2 antibodies detected PrP^res^ equally well in extracts from persons with sCJD or with VPSPr. All lanes were loaded with 5 μL of a 10% (wt/vol) brain homogenate. Brain homogenates were analyzed after digestion with Proteinase K. Lane 1, molecular weight marker (sizes indicated at left in kDa); lane 2, sCJD MM1; lane 3, VPSPr UK-VV; lane 4, VPSPr UK-MV; lane 5, VPSPr NL-VV; lane 6, additional VPSPr of UK origin; lane 7, sCJD VV2. MM, homozygous for methionine; PrP^res^, protease-resistant isoform of the disease-specific prion protein; NL-VV, patient from the Netherlands who had VPSPr and the codon 129VV genotype; sCJD, sporadic Creutzfeldt-Jakob disease; UK-VV and UK-MV, patients from the United Kingdom who had VPSPr and the codon 129VV and 129MV genotypes, respectively; VPSPr, variably protease-sensitive prionopathy; VV, homozygous for valine.

Increased sensitivity in the detection of PrP fragments by Western blotting using the monoclonal antibody 1E4 has been reported in cases of VPSPr (*4*,*10*). In this study, a direct comparison of the detection sensitivity of 2 PrP antibodies (monoclonal antibodies 1E4 and 3F4) was carried out by Western blot by using brain homogenate from all 3 VPSPr patients in the transmission series. Consistent with our previous findings (*5*,*6*), we found no increase in the PrP detection sensitivity by using 1 antibody or the other (Figure 2).

### Absence of Clinical Disease and Vacuolar Pathology

No evidence of clinical disease with vacuolar pathology was observed in any of the 133 mice inoculated in this transmission series (Table 2). Furthermore, no vacuolar pathology was observed in any of the asymptomatic mice in the study. Clinical neurologic signs were observed on a few occasions without pathologic confirmation of prion disease, suggesting a nontransmissible spongiform encephalopathy condition related to the age of the mice in the study.

**Table 2:** Results of intracerebral inoculation of brain tissue homogenates from 3 patients with variably protease-sensitive prionopathy into 3 lines of human transgenic mice*

Brain inoculum source, mouse line†	No. mice positive/no. total			Mean no. PrP plaque–like deposits (range)¶
	Clinical signs of prion disease‡	Vacuolar degeneration‡	PrP deposition§	
UK-VV				
HuMM	0/15	0/15	1/15	0#
HuMV	4/15	0/15	2/14	5 (2–8)
HuVV	0/14	0/14	5/14	10 (1–17)
UK-MV				
HuMM	1/15	0/15	0/15	0
HuMV	1/15	0/15	0/15	0
HuVV	0/15	0/15	0/15	0
NL-VV				
HuMM	0/15	0/15	0/15	0
HuMV	0/15	0/15	3/15	8 (1–15)
HuVV	0/14	0/14	7/14	3 (2–4)

*HuMM, HuMV, and HuVV, transgenic mice expressing the different forms of the human PrP gene (i.e., those homozygous for methionine [MM] or valine [VV] or heterozygous for methionine and valine [MV]); PrP, prion protein. †Brain inoculum was prepared from postmortem samples from persons with variably protease-sensitive prionopathy. NL-VV, patient from the Netherlands who had the PrP codon 129VV genotype; UK-MV, patient from the United Kingdom who had the PrP codon 129MV genotype; UK-VV, patient from the United Kingdom who had the PrP codon 129VV genotype. ‡In mice with a positive score for clinical signs of a prion disease and a negative score for vacuolar pathology, the neuropathologic assessment was considered definitive. §A positive score for PrP pathology was given to mice showing PrP deposition in the brain with at least 1 of the 4 PrP antibodies used in the immunohistochemical analysis. ¶The number of plaque-like deposits was counted per mouse, and results are given as mean (range) for each genotype. #Mouse showed evidence of PrP deposition in a tumor.

### Minimal PrP Deposition in Restricted Brain Regions

**Figure 3:**
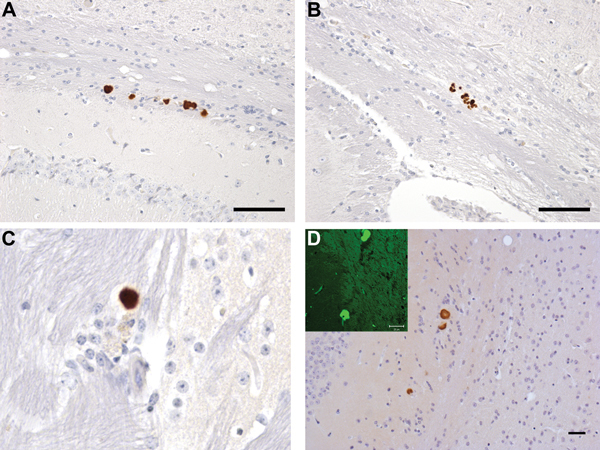
Neuropathology in transgenic mice following inoculation with brain homogenate prepared from a postmortem sample from a person with VPSPr. Numerous PrP-labeled plaque-like deposits within the corpus callosum of HuVV (A) and HuMV (B) mice inoculated with brain homogenate from patient UK-VV. C) A single small PrP-labeled plaque in the stratum oriens of the hippocampus following experimental challenge with brain homogenate from patient NL-VV. D) PrP-labeled plaque-like-deposits in the corpus callosum of a HuVV mouse inoculated with brain homogenate from patient UK-VV; inset: Thioflavin-S (Sigma, Gillingham, UK) staining of amyloid, viewed under ultraviolet light in a HuVV mouse challenged with brain homogenate from patient UK-VV. HuVV and HuMV, transgenic mice expressing human PrP gene sequence coding for the valine-homozygous and methionine/valine-heterozygous codon 129 genotypes, respectively; NL-VV and UK-VV, patients from the Netherlands and United Kingdom, respectively, who had VPSPr and the valine-homozygous codon 129 genotype; PrP, prion protein; VPSPr, variably protease-sensitive prionopathy. Scale bars indicate 25 μm.

Immunohistochemical analysis for PrP in mice challenged with brain homogenate prepared from VPSPr patient UK-MV showed no evidence of PrP accumulation within the brain of inoculated mice. Immunohistochemical analysis for PrP in mice challenged with homogenate prepared from the brain of patient NL-VV showed PrP accumulation in 7 of 14 HuVV and 3 of 15 HuMV transgenic mice, but no PrP deposition was found in the HuMM mice (Table 2). PrP deposits were detected most frequently with the PrP antibodies 6H4 and 3F4 and less frequently with antibodies KG9 and 12F10. This differential labeling is similar to that reported in human cases of VPSPr and may be related to the conformation of VPSPr-associated PrP and the availability of the epitopes that the antibodies detect (*5*,*17*,*20*). The pattern of PrP accumulation was limited to small, often numerous, focal plaque-like deposits located within the corpus callosum and the stratum oriens and stratum lacunosum moleculare of the hippocampus and parallel to the lateral ventricle (Figure 3, panels A–C). Sections treated with Thioflavin-S confirmed that these plaque-like deposits in the corpus callosum and its vicinity were composed of amyloid (Figure 3, panel D).

**Figure 4:**
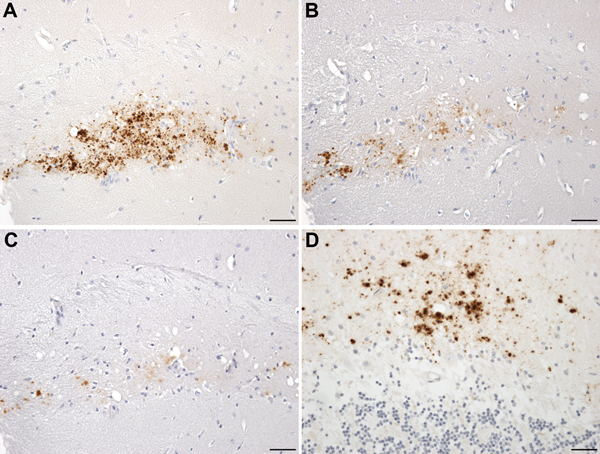
Neuropathology in transgenic mice following inoculation with brain homogenate prepared from a postmortem sample from a person with VPSPr. A–C) Immunohistochemistry for the PrP, showing small granular and microplaque-like deposits within the CA3 region of the hippocampus of a HuVV mouse challenged with VPSPr inoculum prepared from patient UK-VV. Differential staining was observed in this mouse by using the following PrP antibodies: Purified (3F4) (Cambridge Bioscience, Cambridge, UK) (A); Prion Protein Monoclonal Antibody (12F10) (Bioquote Ltd, York, UK) (B); and mAb 6H4 (Prionics AG, Schlieren, Switzerland) (C). D) Molecular layer of the cerebellum of a person with VPSPr, showing microplaque deposits stained with monoclonal antibody 3F4. HuVV, transgenic mouse expressing human PrP gene sequence coding for the valine-homozygous codon 129 genotype; PrP, prion protein; UK-VV, patient from the United Kingdom who had VPSPr and the valine-homozygous codon 129 genotype; VPSPr, variably protease-sensitive prionopathy. Scale bars indicate 25 μm.

In mice challenged with a homogenate prepared from the brain of patient UK-VV, 5 of 14 HuVV, 2 of 14 HuMV, and 1 of 15 HuMM mice showed evidence of PrP deposits (Table 2). HuVV and HuMV mice showed PrP deposits similar to those found in the mice challenged with extract prepared from the brain of patient NL-VV, with 1 exception: a single HuVV mouse showed plaque-like accumulations and a pattern of intensely stained, small, round granules surrounded by fine target-like punctate staining within the CA3 region of the hippocampus and hippocampal fissure (Figure 4, panels A–C). The larger granular deposits resembled the microplaque accumulations found within the molecular layer of the cerebellum (Figure 4, panel D), hippocampal formation, basal ganglia, and thalamus in humans with VPSPr (*5*). The PrP-positive granules within the hippocampus stained most intensely with antibodies 3F4 and 12F10 and less intensely with antibodies KG9 and 6H4; this finding was similar to that observed in brains of patients with VPSPr (*5*,*6*,*17*) (Figure 4).

### No PrP^res^ Detected by Biochemical Analysis

Western blot analysis was performed on frozen brain tissue from 6 HuVV mice challenged with brain homogenate from VPSPr patient UK-VV. Immunohistochemistry results showed that 4 of the 6 mice had small, plaque-like deposits within the corpus callosum, and 1 of the 4 mice also had microplaque-like deposits in the hippocampus. Neuropathologic examination of the remaining 2 mice showed no evidence of transmission. Four noninoculated HuVV mice were included as negative controls. No disease-specific banding was observed in any of the mice (data not shown); this finding is consistent with the extremely low levels of PrP deposition detected by immunohistochemistry.

### Astrocytic Reactivity Associated with a Single Transmission 

**Figure 5:**
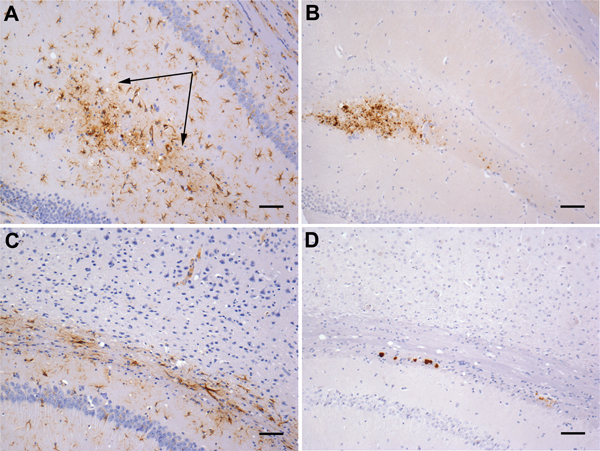
Gliosis in transgenic mice following inoculation with brain homogenate prepared from a postmortem sample from a person with variably protease-sensitive prionopathy. A) Immunohistochemical staining for GFAP in the hippocampus of a HuVV mouse showing microplaque-like deposits. Arrows indicate areas of reactive astrocytosis. B) A serial section from the same HuVV mouse immunolabeled for PrP by using monoclonal antibody (Purified [3F4], Cambridge Bioscience, Cambridge, UK). C) Immunohistochemical staining for GFAP in the hippocampus of a HuVV mouse showing plaque-like deposits. No reactive astrocytosis is seen in the vicinity of plaques. D) A serial section from the same HuVV mouse immunolabeled for PrP by using monoclonal antibody 3F4. GFAP, glial fibrillary acid protein; HuVV, transgenic mouse expressing human PrP gene sequence coding for the valine-homozygous codon 129 genotype; PrP, prion protein. Scale bars indicate 50 μm.

HuVV mice were examined for evidence of astrocytic gliosis. In mice showing only plaque-like deposits of PrP, there was no association between astrocytosis and PrP deposition and no evidence of reactive astrocytosis (Figure 5). In contrast, in the HuVV mice inoculated with brain homogenate from patient UK-VV, a single mouse showed reactive astrocytosis in the vicinity of the microplaque-like deposits (Figure 5). This HuVV mouse is the same mouse that showed evidence of plaque-like and microplaque-like deposits similar to those found in humans with VPSPr.

## Discussion

The inoculation of homogenates prepared from the brains of 3 patients with VPSPr (UK-MV, UK-VV and NL-VV) into transgenic mice expressing the different forms of the human PrP gene has resulted in very different transmission properties when compared with those of previously characterized sCJD strains (*3*). No clinical disease or vacuolar pathology was observed in any of the mice. The only evidence for transmission of disease was the neuropathologic finding of abnormal PrP accumulation in the form of microplaque-like and granular deposits in the hippocampus and subventricular areas of the brain. These results contrast considerably with those observed with sCJD in the same HuMM/HuMV/HuVV mouse lines (*3*). Sporadic CJD transmits to all these mouse lines, as indicated by evidence of clinical signs and vacuolar pathology and/or PrP deposition, and the combination of these transmission properties has resulted in the identification of 4 strains of sCJD (*3*). Of the 4 sCJD strains, subtype VV2 showed the greatest frequency of clinical (13/16), vacuolar (16/16), and pathologic (15/15) signs of prion disease following inoculation into HuVV mice (Table 3) (*3*). In contrast, the MM2 subtype of sCJD showed the least transmission to the mice: no mice had clinical signs or vacuolar pathology, and only 3 of 17 HuVV and 2 of 18 HuMV mice had evidence of PrP deposition in the form of small punctate deposits in the thalamus (Table 3) (*3*). Although this transmission of the MM2 subtype might be considered similar to that of VPSPr, the PrP deposition differed in form and brain area compared with the deposition observed in the VPSPr studies. Unlike sCJD, which shows transmission from patients with all 3 codon 129 genotypes, only VPSPr from the 2 patients with codon 129VV provided evidence of transmission. This low rate of transmission may be due to low levels of PrP^res^ in the brain homogenates that were inoculated, or it could be that the PrP genotype plays a role in transmission of disease.

**Table 3:** Results of intracerebral inoculation of brain tissue homogenates from 4 patients with different subtypes of the sporadic form of Creutzfeldt-Jakob disease into 3 lines of human transgenic mice*

Brain inoculum source, mouse line†	No. mice positive/no. total		
	Clinical signs of prion disease	Vacuolar degeneration	PrP deposition
MM1			
HuMM	10/13	13/13	13/13
HuMV	9/14	14/14	14/14
HuVV	8/16	13/16	14/16
MM2			
HuMM	0/16	0/16	0/15
HuMV	0/18	0/18	2/18
HuVV	0/17	0/17	3/17
VV1			
HuMM	0/16	0/16	2/16
HuMV	2/14	9/14	1/14
HuVV	2/14	7/14	7/14
VV2			
HuMM	4/18	6/18	15/17
HuMV	1/15	5/15	12/14
HuVV	13/16	16/16	15/15

*Data adapted from Bishop et al. (*3*). HuMM, HuMV, and HuVV, transgenic mice expressing the different forms of the human PrP gene (i.e., those homozygous for methionine [MM] or valine [VV] or heterozygous for methionine and valine [MV]). PrP, prion protein. †Brain inoculum was prepared from postmortem samples from persons who had the MM1, MM2, VV1, or VV2 subtype of sporadic Creutzfeldt-Jakob disease.

Prion disease propagation involves the aggregation of abnormal PrP that acts as a template for further aggregation within the brain, a process termed seeding (*21*,*22*). The spread of PrP within the brain appears to occur in cell-to-cell fashion in well-defined neuroanatomic pathways (*23*), the mechanisms of which are yet to be elucidated despite extensive studies. Prion diseases have the potential to be transmissible between persons, a fact that raises public health concerns, particularly regarding vCJD. Assessing the risk for transmission is a challenge because of the varied nature of prion diseases and conflicting evidence over the mechanisms of transmission. Risk assessment is made even more complicated by the existence of prion disease models in which negligible amounts of PrP^res^ are associated with high infectivity titers in vivo (*24*) and also of models in which PrP^res^ in the form of amyloid plaques develops in the absence of clinical disease or spongiform changes (*25*).

It could be argued that the observation of small plaque-like amyloid deposits in the brains of mice with no neurologic signs of disease after the inoculation of brain homogenates prepared from patients with VPSPr does not indicate disease transmission. Instead, the deposits could indicate an amyloid seeding phenomenon akin to that observed following the experimental inoculation of primates with brain tissue from patients with Alzheimer disease (*26*). In those experiments, amyloid β seeding occurred in the primate brain in the absence of any clinical signs. Precedence of this phenomenon in prion disease has been set by Piccardo et al. (*27*), who showed similar results in a mouse model system of prion disease transmission. However, in our study, the brain of 1 mouse exhibited intensely stained, small, round granules within the hippocampus in addition to the plaque-like deposits (Figure 4). These small granules are reminiscent of the microplaques found in brain tissue of humans with VPSPr (*4*,*5*). Furthermore, with 4 PrP antibodies, the microplaque deposits in the mouse brain showed the same pattern of differential immunoreactivity as that in the brain of patients with VPSPr (*5*,*6*,*17*). Moreover astrocytosis in the vicinity of the microplaques was also observed in this mouse (Figure 5). This type of astrocytic response is observed in all our model systems of transmissible prion disease, but is absent from the nontransmissible forms of PrP (i.e., amyloid plaques in absence of clinical disease), suggesting that this single mouse may represent a transmission of infection rather than a consequence of seeding of inoculum (*25*,*28*). Second passage in the same mouse line will be required to prove this interpretation, but such a study will take an additional 3 years to complete.

Although understanding the mechanisms of transmission is an interesting facet of this study, our primary finding is that VPSPr is capable of transmission to transgenic mice expressing PrP, albeit at extremely low levels compared with those of other transmissible prion diseases (e.g., sCJD and vCJD). We demonstrate that VPSPr is a disease with biological properties distinct from those of sCJD and with a limited, but not negligible, potential for infectivity. These results demonstrate the importance of continuing surveillance to fully uncover the growing spectrum of human prion diseases.
